# Clinical outcome and pathologic correlation of stereotactic body radiation therapy as a bridge to transplantation for advanced hepatocellular carcinoma: a case series

**DOI:** 10.1186/s13014-020-01739-5

**Published:** 2021-01-14

**Authors:** Ying-Fu Wang, Yang-Hong Dai, Chun-Shu Lin, Hao-Chih Chang, Po-Chien Shen, Jen-Fu Yang, Chih-Weim Hsiang, Cheng-Hsiang Lo, Wen-Yen Huang

**Affiliations:** 1 Department of Radiation Oncology, Tri-Service General Hospital, National Defense Medical Center, No. 325, Cheng-Gong Rd, Neihu, Taipei, 114 Taiwan; 2 Department of Radiology, Tri-Service General Hospital, National Defense Medical Center, No. 325, Cheng-Gong Rd, Neihu, Taipei, 114 Taiwan

**Keywords:** Hepatocellular carcinoma, Liver transplantation, Radiotherapy, Stereotactic body radiotherapy

## Abstract

**Background:**

Stereotactic body radiotherapy (SBRT) is an emerging modality for hepatocellular carcinoma (HCC). However, there is scant information about its safety and effectiveness in the neoadjuvant setting prior to liver transplantation (LT). We present the clinical outcome and pathologic assessment of SBRT followed by LT for patients with advanced HCC.

**Methods:**

This retrospective study included HCC patients treated with neoadjuvant SBRT prior to LT between 2009 and 2018. Radiographic response and adverse effects, including radiation-induced liver disease (RILD), were evaluated. Pathologic response was assessed by the percentage of tumor necrosis relative to the total tumor volume. Overall survival (OS) and recurrence-free survival (RFS) were calculated using the Kaplan–Meier method.

**Results:**

Fourteen patients underwent SBRT for a total of 25 HCC lesions, followed by LT. The median tumor size was 4.45 cm in diameter, and the median prescribed dose was 45 Gy in 5 fractions. SBRT provided significant AFP reduction, 100% infield control, and a 62.5% response rate. The maximum detected toxicity included grade 3 thrombocytopenia and two grade 3–4 hyperbilirubinemia. One patient developed non-classic RILD. Patients were bridged to LT with a median time of 8.4 months after SBRT, and 23.1% of them achieved a complete pathologic response. The median OS and RFS were 37.8 and 18.3 months from the time of LT, respectively.

**Conclusions:**

SBRT provides favorable tumor control and acceptable adverse effects for patients awaiting LT. Further prospective studies to test SBRT as a bridging therapy for LT are feasible.

## Background

Hepatocellular carcinoma (HCC) is the most common primary hepatic malignancy and a leading cause of cancer mortality worldwide [1]. Most HCCs are caused by chronic liver disease; for instance, HCCs among Asians are caused by chronic viral hepatitis. Liver transplantation (LT) is a potentially curative therapy for HCC and is the best option for patients with cirrhosis and portal hypertension. The Milan criteria and the expanded set of criteria proposed by the University of California San Francisco (UCSF criteria) are well-validated guidelines for LT [2, 3]. Through tumorous liver removal and liver function correction, LT provides excellent local control and leads to a 4-year post-transplant survival rate of 85% and recurrence-free survival rate of 92% [2, 4].

Unfortunately, only a small percentage of HCC patients could receive transplants due to the scarcity of donors and the great number of nonmalignant indications. Furthermore, HCC patients are at risk of tumor progression, making them ineligible for transplantation that involves a long waiting period, which further results in a higher dropout rate compared to nonmalignant candidates (31.8% vs. 19.1% at 1 year) after listing for LT [5]. Various locoregional therapies serve as a bridging strategy that aim to prevent the dropping out of waitlisted patients, or as a downstaging strategy to convert advanced HCC to LT candidates. Transarterial chemoembolization (TACE) and radiofrequency ablation (RFA) have been the most commonly used approaches in controlling tumor growth and vascular invasion, with a reportedly low dropout rate of 12.2% [6].

Historically, hepatic radiotherapy was limited due to the low radiation tolerance of the liver and the potential for radiation-induced liver disease (RILD). With precise delivery of an ablative dose of radiation to the target tumor within a limited number of fractions, stereotactic body radiotherapy (SBRT) has been developed as a safe and effective locoregional therapy for both primary and metastatic hepatic neoplasms [7]. SBRT was reported to achieve a 2-year local control rate of 74–100% and overall survival rate of 34–68.7% that are comparable with outcomes after RFA and TACE [8]. However, the effectiveness and safety of SBRT in the neoadjuvant setting are still under investigation [9–17], and information on clinical outcomes and pathological responses after irradiation are still scarce. Here, we report the clinical outcomes of neoadjuvant SBRT followed by LT for patients with advanced HCC, as well as the pathologic evaluation of HCC lesions treated with radiotherapy.

## Methods

We retrospectively reviewed the medical records of HCC patients treated with neoadjuvant SBRT in our institution from January 2009 to December 2018. The inclusion criteria were as follows: (1) patients with confined HCC without extrahepatic metastases, (2) an Eastern Cooperative Oncology Group performance status of ≤ 2, and (3) no previous abdominal radiotherapy. Pathologic diagnosis of HCC was not required as long as established radiographic criteria were satisfied for diagnosis [18]. SBRT was used in bridging or downstaging prior to LT. Other prior liver-directed therapies for HCC have been allowed. Our institutional review board (IRB) approved this study and waived the requirement for informed consent owing to the retrospective nature of the study.

### SBRT planning and treatment

All patients were immobilized using a vacuum cushion in the supine position during simulation and treatment. Contrast enhanced computed tomography (CT) simulation with 3 mm slice thickness was performed, and previous dynamic CT or magnetic resonance imaging (MRI) were used as a reference to determine disease extent.

For patients with multiple tumors and preserved liver function, we would like to treat all lesions with SBRT as possible with respect to the dose constraints of the critical organs, especially the normal liver. If the SBRT plan could not cover all lesions, we would like to target the portal vein tumor thrombosis (PVTT) and the index tumor. The gross tumor volume (GTV) was defined as an enhancing tumor seen on CT and/or MRI. The planning target volume (PTV) was created by expanding the 0- to 8-mm margin around the GTV. Modification of the PTV was acceptable when overlapping the dose-limiting organs, except for the normal liver. The radiation dose was prescribed to PTV individually based on the normal organ dose constraints determined by the institutional protocol.

SBRT was delivered using either the CyberKnife system (Accuray Inc., Sunnyvale, CA) or TomoTherapy system (Accuray Inc., Sunnyvale, CA). Patients treated using CyberKnife were treated with respiratory tracking of the tumor via peritumoral fiducials. For patients treated using TomoTherapy, breathing motion management was conducted with abdominal compression to reduce liver motion and with four-dimensional CT images to estimate internal target volume.

### Response and toxicity evaluation

All patients were assessed during the entire course of SBRT, at 1- to 3-month intervals after completion of SBRT until orthotopic LT, at 3- to 4-month intervals for the first 2 years after surgery, and at 6-month intervals thereafter. Clinical evaluation, complete blood count, liver function, Child–Pugh score, alpha-fetoprotein (AFP), toxicity, and imaging with either contrast-enhanced dynamic CT or MRI were performed. Radiographic response to SBRT was assessed according to the modified Radiographic Evaluation Criteria in Solid Tumors (mRECIST) [19]. In-field failure was defined as disease progression or new enhancement within the PTV or at its margins. For patients with AFP elevation (≥ 20 IU/mL) before SBRT, reduction in AFP was calculated using the pretreatment baseline minus the minimal value after irradiation, and was censored at the time of intrahepatic progression, liver-directed therapies, or transplant. Acute and late adverse effects were graded using the National Cancer Institute Common Terminology Criteria for Adverse Events v4.0. RILD was established in the absence of intrahepatic disease progression (based on mRECIST criteria) within 3 months after completion of SBRT, and was recorded in two distinct entities. Classic RILD includes anicteric hepatomegaly, nonmalignant ascites, and elevated alkaline phosphatase of at least twice the upper normal limit. The non-classic RILD is composed of liver transaminase elevation of more than 5 times the reference value, or worsening of liver metabolic function represented as an increase of 2 or more points in the Child–Pugh score. For patients with preexisting abnormal transaminase and/or alkaline phosphatase, the pretreatment baseline was adopted rather than the upper limits of the normal range. Patients were reassessed for transplant candidates using the Milan criteria, and underwent either deceased donor liver transplantation (DDLT) or living donor liver transplantation (LDLT), as clinically appropriate.

After the LT, we evaluated the SBRT treatment effect through pathological assessment. The pathologic response was estimated as the percentage of tumor necrosis relative to the total tumor volume, as follows: complete pathologic response was 100% tumor necrosis and the absence of any viable tumor; significant pathologic response was 50–99% tumor necrosis in cross-section; minimal pathologic response was 1–49% tumor necrosis; and no pathologic response was no tumor necrosis present [11]. The correlation between pathological response and radiologic response based on the mRECIST criteria was analyzed using the Pearson correlation coefficient. Disease recurrence, actuarial overall survival (OS), and recurrence-free survival (RFS) were analyzed from the time of transplantation using the Kaplan–Meier method.

## Results

### Patients

**Fig. 1:**
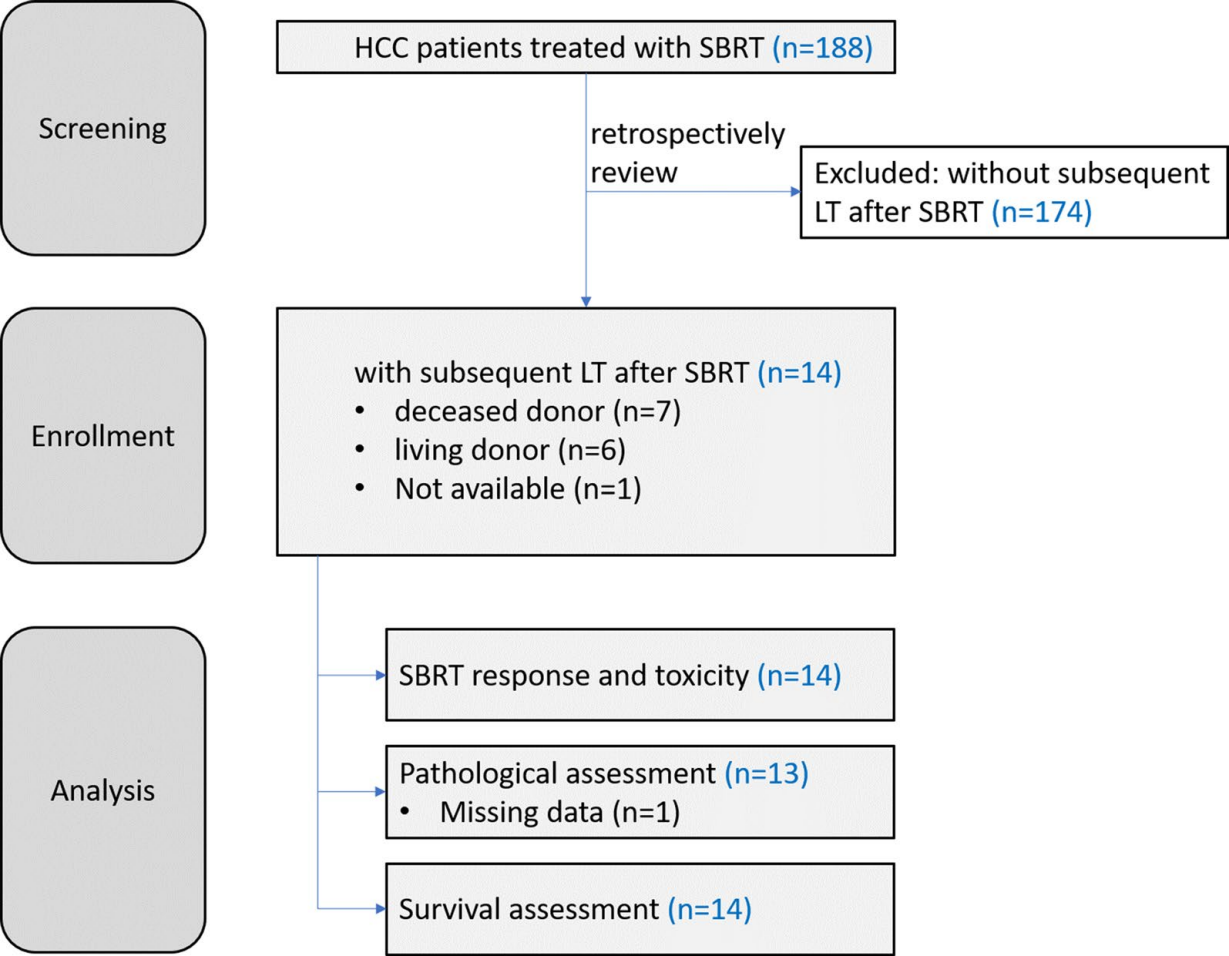
Consort diagram of the study protocol. We retrospectively screened 188 patients who received SBRT at our institution between 2009 and 2018. Fourteen patients underwent liver transplantation, and they were included in this study

We retrospectively screened 188 patients who received SBRT at our institution between 2009 and 2018. Fourteen patients were included in this study (Fig. 1), and the baseline characteristics at the time of SBRT are listed in Table 1. The median age was 55.5 years, and most patients had underlying hepatitis B virus infection (78.6%) and preserved liver function (Child–Pugh class A, 78.6%). Thirteen patients have received prior liver-directed treatment for HCC in the form of TACE alone or TACE combined with other modalities. Ten (71%) patients had multifocal disease and 4 (28.7%) was found to have PVTT. Four (28.6%) patients met the Milan criteria for LT at the time of SBRT.

**Table 1:** Patient characteristics at the first time of SBRT (n = 14)

Characteristics	
Age, median (range)	55.5 (40–68)
Male, no. (%)	12 (85.7)
Cause of cirrhosis, no. (%)	
HBV	11 (78.6)
HCV	2 (14.3)
Alcoholic	1 (7.1)
ECOG performance status, no. (%)	
0	9 (64.3)
1	4 (28.6)
2	1 (7.1)
Child–Pugh class, no. (%)	
A	11 (78.6)
B	2 (14.3)
C	1 (7.1)
Previous treatment, no. (%)	
TACE	7 (50.0)
RFA + TACE	1 (7.1)
TACE + Thaldo	2 (14.3)
Segmentectomy + TACE	2 (14.3)
Segmentectomy + TACE + RFA	1 (7.1)
None	1 (7.1)
Max. tumor diameter, median (range)	4.45 (0.9–10.5)
Number of tumors, no. (%)	
Solitary	4 (28.6)
Multiple	10 (71.4)
Portal venous tumor thrombosis, no. (%)	4 (28.6)
AFP, median (range), IU/mL	59.5 (3.0–1286.0)
BCLC stage at SBRT, no. (%)	
A	3 (21.4)
B	6 (42.9)
C	4 (28.6)
D	1 (7.1)
Within Milan criteria before SBRT, no. (%)	4 (28.6)

AFP, alpha-fetoprotein; BCLC, Barcelona clinic liver cancer; RFA, radiofrequency ablation; SBRT, stereotactic body radiation therapy; TACE, transcatheter arterial chemoembolization

Sixteen SBRT courses with either CyberKnife (n = 15) or TomoTherapy system (n = 1) have been conducted for a total of 25 HCC lesions. Two patients underwent repeated SBRT for intrahepatic recurrence between the first SBRT and LT. The SBRT treatment parameters and outcomes are shown in Table 2. The median prescribed dose was 45 Gy (range: 28–60 Gy) in 5 fractions (range: 4–5). The median volumes of treated tumors and the normal liver were 35.9 mL and 1281.7 mL, respectively. Patient no. 7, a 55-year-old man, received a second SBRT due to intrahepatic out-field recurrence 12 months after the first SBRT. He underwent LDLT 11 months after the completion of the second SBRT. Patient no. 10 was a 58-year-old woman who experienced repeated intrahepatic recurrence, and she had been treated with wedge resection, 4 courses of TACE, 2 courses of SBRT (with 32 months interval), and 2 courses of RFA without major adverse events. Eventually, she underwent DDLT 40 months after finishing the second SBRT (8 years after HCC diagnosis).

**Table 2:** SBRT parameters, outcomes, and adverse events (n = 16)

SBRT treatment, median (range)	
SBRT dose, Gy	45.0 (28–60)
Number of fractions	5 (4–5)
EQD2, Gy	71.25 (36.4–110.0)
Tumor volume, cc	35.9 (1.11–819.43)
Number of targeted lesions	2 (1–4)
Normal liver volume, cc	1281.7 (537.0–2095.1)
rV15* of uninvolved liver, cc	1019.6 (436.6–1610.2)

SBRT outcomes, no. (%)	
Radiographic response	
Complete response (rCR)	4 (25)
Partial response (rPR)	6 (37.5)
Stable disease (rSD)	6 (37.5)
Local recurrence	
Out-field	11 (68.8)
In-field	0
AFP after SBRT**, median (range), IU/mL	38.3 (2.8–386.3)
Within Millan criteria after SBRT	5 of 14 patients (35.7)

Adverse events, no. (%)	Grade 1	Grade 2	Grade3	Grade4
Biochemical				
Albumin	3 (18.8)	3 (18.8)	0	0
Alkaline phosphatase	1 (6.3)	0	0	0
ALT	7 (43.8)	0	0	0
AST	7 (43.8)	0	0	0
Bilirubin	0	1 (6.3)	1 (6.3)	1 (6.3)
Hematologic				
Anemia	3 (18.8)	1 (6.3)	1 (6.3)	0
Leukocytopenia	6 (37.5)	1 (6.3)	1 (6.3)	0
Thrombocytopenia	5 (31.3)	1 (6.3)	3 (18.8)	0
Fatigue	2 (12.5)	0	0	0
Gastrointestinal toxicity				
Abdominal pain	2 (12.5)	0	0	0
Anorexia	1 (6.3)	0	0	0
Diarrhea	1 (6.3)	1 (6.3)	0	0
Nausea/vomiting	1 (6.3)	2 (12.5)	0	0

RILD, no. (%)	
Classic	0 (0)
Nonclassic	1 (6.3)

AST, aspartate aminotransferase; ALT, alanine aminotransferase; rCR, radiological complete response; rPR, radiological partial response; rSD, radiological stable disease; RILD, radiation induced liver disease; SBRT, stereotactic body radiation therapy

*rV15 was defined as volume of uninvolved liver receiving ≤ 15 Gy (cc)

**Only apply for patients with elevated AFP level at baseline

There were 10 patients with treatment failure before LT, all in the form of intra-hepatic out-field failure. For out-field recurrence after SBRT, 8 patients received further salvage treatments (median, 1; range, 0–6 times) prior to LT, and 2 patients underwent LT without additional anti-HCC treatment. The first salvage treatment included SBRT (n = 1), TACE (n = 3), RFA (n = 1), multi-kinase inhibitor (n = 1), and TACE combined with a multi-kinase inhibitor (n = 2). Repeated intrahepatic out-field recurrence was noted after salvage treatment in 4 patients, and combined treatment modalities were arranged before LT.

### Response to SBRT as a bridge therapy

Complete radiologic response was observed in 4 (25%) of the 16 SBRT courses, partial response in 6 (37.5%), and stable disease in 6 (37.5%), with an infield control rate of 100% reached prior to LT (Table 2). For the 7 patients with pretreatment elevated AFP (median: 148.0 IU/mL), maximal AFP response (median: 38.3 IU/mL) was observed at 1.5 months (range: 0.8–5.1 months) after the first day of SBRT. After completion of SBRT, 1 of the 4 patients within the Milan criteria initially had intrahepatic out-field tumor progression and was dropped out. Among the 10 patients initially beyond the Milan criteria, 2 had tumors that shrunk and met transplant eligibility after SBRT.

All 14 patients completed the prescribed courses of SBRT. The most common adverse effects were grade 1–2 gastrointestinal toxicities. Three (18.8%) patients developed grade 3 thrombocytopenia, and 2 (12.5%) patients had grade 3 or 4 blood hyperbilirubinemia (Table 2). Non-classic RILD developed in 1 patient (patient no.8), who had alcoholic cirrhosis and HCC after TACE, with common hepatic duct invasion and PVTT. He received SBRT to the residual tumor at 35 Gy in 5 fractions, and the volume of the uninvolved liver that received ≤ 15 Gy was 654 mL. At 2 months after irradiation, acute suppurative cholangitis and non-classic RILD (Child–Pugh A6 to C12) developed. The hepatic toxicity recovered partially to Child–Pugh C10 after his cholangitis was controlled, and he underwent DDLT 8.6 months after SBRT.

Subsequently, all patients successfully underwent LT from deceased donors (n = 7) or living donors (n = 6). Patient no. 13 underwent LT in mainland China, with the medical records of the donor type as well as the pathology report both unavailable. The median duration from the time of last SBRT to LT was 8.4 months (range: 1.6–62.4 months). There were no major perioperative complications.

Details regarding the 13 patients with evaluable surgical pathology are listed in Table 3. Twelve patients had residual HCC, of which 11 had multifocal tumors. Three patients had complete pathologic response, 3 had significant pathologic response, and 5 had minimal pathologic response. Information about tumor necrosis were insufficient in 2 patients. Poor concordance was observed, with a Pearson correlation coefficient of − 0.434 between the percentage of necrosis and the post-SBRT radiologic response according to the mRECIST criteria.

**Table 3:** Pathological response to SBRT and the correlation between clinical response

Patient	Pre-LT SBRT							LT						
	BCLC stage	Dose (Gy)/fraction number	Prescription isodose/maximum dose*	Radiological response	RILD	Milan criteria	Median time to LT (months)	Donor type	Pathologic AJCC 8th stage	Max. tumor size	PVTT	Tumor necrosis	Recurrence site	Status
1	B	42/5	75%/5600 cGy	SD	–	B → B	1.6	CLT	IIIA	2.5	Present	30%	–	DOO
2	B	60/5	72%/8333 cGy	PR	–	B → B	34.3	LDLT	II	2.2	–	5%	Liver, carcinomatosis	DOD
3	B	45/5	75%/6000 cGy	SD	–	B → B	7.3	LDLT	II	2.5	–	100%	Liver	DOD
4	B	40/5	70%/5714 cGy	SD	–	B → B	5.8	LDLT	II	6	–	99%	–	NED
5	A	50/5	80%/6250 cGy	PR	–	W → W	17.0	CLT	II	2.5	–	100%	–	NED
6	C	55/5	73%/7534 cGy	PR	–	B → B	4.0	LDLT	II	3.5	Present	10%	Bilateral lung	DOD
7a b	A B	45/5 45/5	79%/5696 cGy 75%/6000 cGy	CR CR	– –	W → W W → B	23.0	LDLT	II	1	–	5%	–	DOO
8	C	35/5	72%/4861 cGy	CR	Nonclassic	B → W	8.6	CLT	IIIA	6	–	90%	–	DOO
9	A	40/4	79%/5063 cGy	PR	–	W → W	62.4	CLT	IIIB	5.5	Present	NA	–	DOO
10a b	B 0	50/5 50/5	78%/6410 cGy 76%/6579 cGy	PR SD	– –	B → W W → W	40.5	CLT	IIIC	3.5	–	95%	liver, lymph nodes, left adrenal gland,	DOD
11	C	45/5	75%/6000 cGy	PR	–	B → B	11.7	LDLT	I	6.5	–	30%	–	DOO
12	C	47.5/5	72%/6690 cGy	SD	–	B → B	2.1	CLT	No viable tumor	–	–	–	–	NED
13	B	28/5	76%/3683 cGy	SD	–	B → B	6.2	NA	NA	NA	NA	NA	Solitary lung	NED
14	D	50/5	90%/5853 cGy	CR	–	W → W	8.3	CLT	IA	1.5	Present	NA	–	NED

B, beyond Milan criteria; BCLC, Barcelona clinic liver cancer; CLT, cadaveric liver transplantation; CR, complete response; DOD, died of disease; DOO, dead of other causes; LDLT, living donor liver transplantation; LT, liver transplantation; NA, not available; NED, no evidence of disease; PR, partial response; PVTT, portal vein tumor thrombosis; RILD, radiation-induced liver disease; SD, stable disease; W, within Milan criteria

### Outcome after LT

**Fig. 2:**
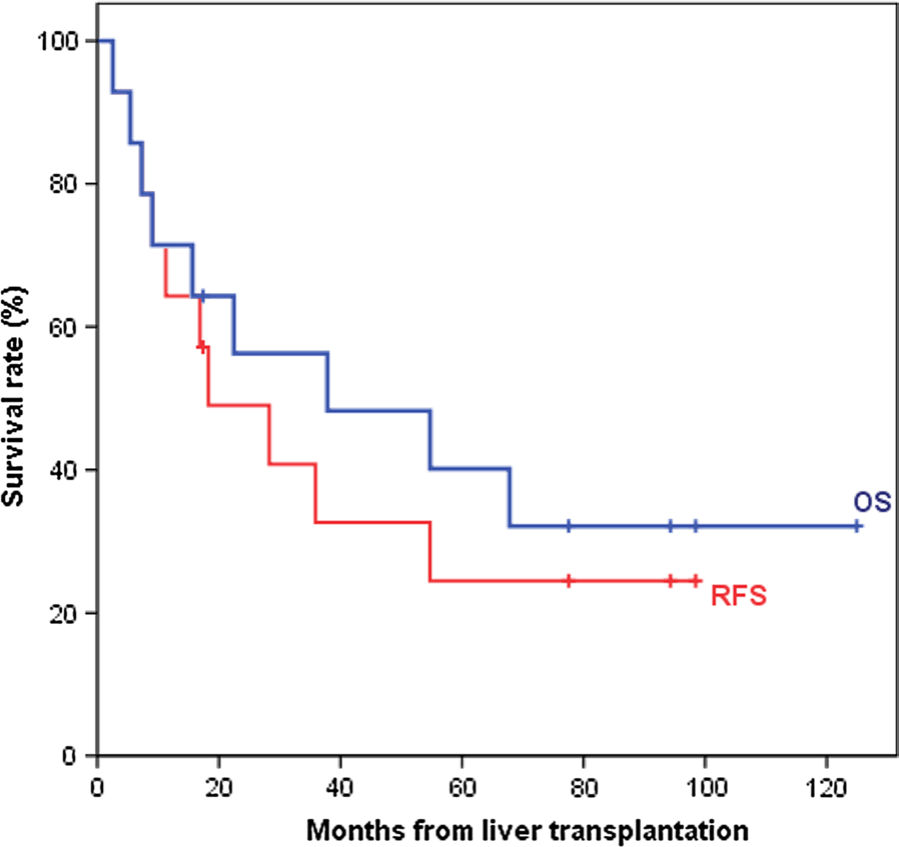
Kaplan–Meier survival curves for overall survival (OS) and recurrence-free survival (RFS) in patients with HCC treated with neoadjuvant SBRT and LT. The median OS and RFS were 37.8 and 18.3 months, respectively

The data were collected through June 2020 and the median follow-up from the time of transplantation was 30.2 months (range: 3.2–122.2 months). Five patients were alive during the last follow-up. The median OS was 37.8 months (95% CI: 0–91.96 months), and the median RFS was 18.3 months (95% CI: 0–36.91 months) (Fig. 2). During the follow-up period after transplantation, five patients experienced recurrence (1 with hepatic recurrence and 4 with distant metastasis), and 4 of them died from the disease. Patient no. 13 developed solitary pulmonary metastasis 28 months after LT, and he survived with no evidence of disease after video-assisted thoracoscopic surgery with wedge resection.

The prognostic factors for recurrence-free survival and overall survival were analyzed. However, none of the factors had a significant survival impact in univariate analysis (Additional file 1: Table S1).

## Discussion

This single institutional retrospective study showed that SBRT is an effective and safe modality in neoadjuvant settings even in advanced HCC. After SBRT, patients smoothly underwent LT with favorable pathological responses.

Several liver-directed therapies have been used as a neoadjuvant treatment to prevent HCC patients from dropping out of the transplant waiting list. Fisher et al. reported that aggressive multi-modality therapy with TACE and RFA might optimize the use of LT; this approach had a low dropout rate of 12.2% during a median waiting time of 9.1 months [6]. However, each modality is limited by the tumor size, number, and location, the adjacent critical organs, the liver function, and the patient’s general condition. SBRT has been developed as an effective noninvasive bridge treatment with excellent tumor control and tolerable side effects (Table 4) in several observational studies [9–16], with doses of 30–54 Gy in 3–6 fractions. SBRT provides a low dropout rate and achieves a complete necrosis rate of 27–61.5% of the treated lesions, resulting in favorable overall and disease-free survival after LT.

**Table 4:** Summary of literature on radiation therapy as a bridge to transplantation in HCC

Study, year(ref.)	Pre-operative radiotherapy					Liver transplantation				
	No. of patients /lesions	Child–Pugh class	Dose (Gy)/no. of fractions	Infield control rate (%)	Toxicity ≥ grade 3/RILD	No. of patient	Median time to LT (months)	Pathologic response	Post-transplant outcome	Median follow-up (months)
Sandroussi, 2010 [8]	10/10	A: 40% B: 50% C: 10%	33/1–6	100%	0/0	5	5.2	SpR: 3 (60%) MpR: 1 (20%) NpR: 1 (20%)	6-month RFS: 70%	6
Andolino, 2011 [9]	60/NA	A: 60% B: 40%	40–44/3	NA	21/NA	23	7	NA	2-year OS: 96% 2-year PFS: 69%	27
Katz, 2011 [10]	18/21	A: 17% B: 44% C: 22% NA:17%	50/10	100%	1/0	11	6	CpR: 2 (18.1%) SpR: 3 (27.2%) MpR 4 (36.6%) NpR: 2 (18.1%)	1 liver recurrence 10 alive with NED	19.6
O'Connor, 2012 [11]	10/11	A: 70% B: 20% C: 10%	51/3	100%	0/0	10	3.8	NpR: 3 (30%)	5-year OS: 100% 5-year DFS: 100%	62
Guarneri, 2016 [12]	8/13	A: 62% B: 38%	36–48/3–5	100%	0/1	8	3.2	CpR: 8 (61.5%) SpR: 1 (7.7%) MpR: 2 (15.3%) NpR: 2 (15.3%)	No liver recurrence 7 alive with NED	9.6
Moore, 2017 [13]	23/NA	A: 57% B: 43%	30/5 48/4 54/3	100%	0/1	11	4.8	CpR: 3 (27.3%) SpR: 6 (54.5%) MpR: 2 (18.2%)	Median OS: not reached Median PFS: not reached	12
Mannina, 2017 [14]	38/51	A: 45% B: 55%	36–45/3–5	NA	NA/NA	38	8.8	CpR: 45%	1, 2, 3, 5-year OS: 92%, 86%, 77%, 73% 1, 2, 3, 5-year DFS: 91%, 85%, 74%, 74%	58
Gresswell, 2018 [15]	12/17	B: 58% C: 42%	40/5	100%	0/0	11	5	CpR: 46%	1,2,3,4-year OS: 91%, 91%, 68%, 57%	40
Sapisochin, 2017 [16]	36/NA	A: 61% B: 39%	36/6	NA	NA/NA	30	5.2	CpR: 4 (13.3%) SpR: 12 (40%) MpR: 11 (36.7%) NpR: 3 (10%)	1,3,5-year OS: 83%, 75%,75%	29.6
Present series	14/26	A: 79% B: 14% C: 7%	45/5	100%	4/1	14	8.4	CpR: 3 (27.3%) SpR: 3 (27.3%) MpR: 5 (45.4%)	Median OS: 37.8 months Median RFS: 18.3 months	30.2

CpR, complete pathologic response; LT, liver transplantation; MpR, minimal pathological response; NA, not available; NpR, no pathologic response; OS, overall survival; PFS, progression-free survival; RFS, recurrence-free survival; RILD, radiation-induced liver disease; SpR, significant pathologic response

Sapisochin et al. [17] from the University of Toronto reported an intention-to-treat analysis comparing SBRT with TACE and RFA as a bridge to LT in a large cohort of 406 HCC patients. The calculated model for end-stage liver disease score was slightly higher for the SBRT group, and only 36% of them were within the Milan criteria preoperatively, whereas 23% in the TACE group and 87.7% in the RFA group were within the Milan criteria. The median prescribed radiation dose was 36 Gy (interquartile range: 30–40 Gy) in 6 fractions. SBRT provided a 16.7% dropout rate from the waiting list, similar to those of patients treated with TACE (20.2%) or RFA (16.8%). Although the 5-year cumulative risk of posttransplant recurrence was better in the RFA group, there was no difference in the 5-year posttransplant survival among the three modalities (75% in SBRT, 69% in TACE, and 73% in RFA group). These comparative data suggested that SBRT can be safely utilized as a bridge to LT in patients with HCC. It may offer advantages when TACE or RFA are not applicable or fail to control the tumor.

By contrast, our cohort was in a relatively advanced stage, with only 28.6% patients initially meeting the Milan criteria. The selection of LT candidates relies mostly on tumor size and number. In principle, Milan criteria were utilised for HCC recipients with deceased donor grafts, and expanded criteria of UCSF were used for living donor LT. For patients with lesions exceeding the Milan criteria, tumor differentiation and cancer-related symptoms were also considered regardless of the tumor size and number, as the so-called extended Toronto criteria [20]. During our study period, the size and number of tumors were not absolute contraindications to LT. However, patients with extrahepatic disease were absolutely excluded from the waiting list. Given that AFP levels > 500 ng/mL were predictive of poor survival outcomes for patients within or beyond the Milan criteria, AFP level was also incorporated for decision-making purposes [20]. In present study, SBRT still provided significant AFP reduction, 100% infield control, and 62.5% response rate, which were similar to the published series [9, 11–14, 16]. Five patients either underwent downstaging or were kept from dropout, and all 14 patients successfully underwent LT with a median duration from SBRT to LT of 8.4 months, which was longer than that reported in other series [9, 11–14, 16, 17]. However, the actuarial dropout rate was unevaluable in this study because we did not enroll all the patients who received SBRT with bridging intent, but only the patients who received SBRT and LT.

In our cohort, 4 (28.6%) patients had PVTT at the time of SBRT. Their PVTT was included in the SBRT target. According to the BCLC guidelines, patients with HCC and PVTT are usually administered systemic therapy with/without palliative locoregional therapies. However, the presence of PVTT is no longer considered an absolute contraindication to LT; LT remains a critical part of the treatment algorithm [21–23]. In our institute, SBRT is part of the multimodality approach; properly selected patients will be treated with SBRT to the PVTT and index lesion(s). After SBRT, LT could be reconsidered individually at a multidisciplinary board for patients who respond to their Vp3/4 or stable disease during the waiting period.

There is considerable variation among the pathologic complete response rates reported in previous studies. Guarneri et al. achieved a 61.5% pathologic complete response after neoadjuvant SBRT [13], whereas in a study by Sapisochin et al., it was only 13.3% [17]. In our cohort, three of the 13 (23.1%) evaluable patients achieved complete tumor necrosis upon pathological analysis. All irradiated lesions had partial or complete pathologic response with shrinkage or necrosis of the tumor.

In contrast to the reportedly good concordance between pre-transplant radiologic response (according to the mRECIST criteria) and degree of necrosis in patients receiving neoadjuvant TACE [24], no strong correlation between radiologic and pathologic response after SBRT was observed in our study. Mannina et al. assessed the correlation between surgical pathology and radiologic scoring criteria in 38 patients treated with SBRT prior to LT [15]. They demonstrated the poor concordance of pathologic response with mRECIST (sensitivity, 90%; specificity, 17%), RECIST (sensitivity 54%, specificity 50%), and European Association for the Study of the Liver (EASL) criteria (sensitivity 83%, specificity 18%). Unlike the immediate post-treatment decreased- or non-enhancement following RFA and TACE, persistent arterial phase hyperenhancement for at least 12 months is common post SBRT and does not necessarily indicate viable neoplasm [25]. In addition to the mRECIST criteria, the AFP level as well as the apparent diffusion coefficient calculated from MRI sequences could improve the assessment of SBRT response and help to determine the LT candidates [26, 27].

The role of radiation therapy in the management of HCC remains to be limited because of concerns about liver toxicity and RILD. Some cases have been reported to have RILD after SBRT prior to LT. Guarneri et al. reported that one patient developed non-classic RILD 1 month after SBRT with 48 Gy in 3 fractions, and he underwent LT 2.2 months later [13]. Moore et al. reported a patient with Child–Pugh B8 who manifested with RILD after SBRT with 30 Gy in 5 fractions and underwent urgent LT due to hepatic decompensation [14]. The possible causative factors included underlying cirrhosis, low normal liver volume, and reirradiation. Nevertheless, irradiation had been well tolerated as a bridging therapy in our study, with minimal grade 3 or higher toxicity. One (6.3%) patient in our cohort experienced non-classic RILD, which was consistent with the rate reported in previous studies (0.0–12.5%). We did not observe significant surgical complications after SBRT in our cohort. However, the precise relationship between preoperative SBRT and transplant complications, such as rejection or biliary stricture, merits further study.

In our study, 1 patient experienced hepatic recurrence and 4 had distant metastases at the end of follow-up. The median OS was 37.8 months from the time of LT, which was worse than the data reported in previous studies [12, 15–17]. The relatively high recurrence rate and modest post-transplant survival in our cohort were probably due to the advanced disease, along with multinodular disease and/or PVTT. All patients were unsuitable or refractory to other locoregional therapies. In addition, the high recurrence rate could also be explained by the fact that many of the patients in our cohort remained beyond the Milan criteria even after SBRT and underwent LT with a living donor. Otto et al. reported that 88% of patients who successfully bridged or downstaged to fit the Milan criteria were recurrence-free in the 5 years post-LT, compared to 55% of those still exceeding the Milan criteria after neoadjuvant TACE [28]. Appropriate LT candidate selection after SBRT is therefore crucial.

The main limitations of our study include its small case number and the heterogeneity of patient characteristics, prior treatments, and radiation dose. In addition, the retrospective study design and potential selection bias are also key limitations, and no conclusion can be drawn about the actuarial dropout rate and outcome compared to other therapies. However, there are limited reports on the pathologic evaluation post SBRT, and our experience contributes to the expansion of knowledge regarding this noninvasive treatment.

## Conclusions

In conclusion, SBRT is safe and effective as a bridging or downstaging therapy for patients awaiting LT for HCC. SBRT provides favorable tumor control and minimal adverse effects and achieves good pathological response and posttransplant survival. These findings suggest that SBRT is a reasonable preoperative option for patients with advanced HCC.

## Supplementary information

**Additional file 1: Table S1:** Prognostic factors influencing RFS and OS after LT using the Cox proportional hazards model.

## Data Availability

All data generated or analyzed during this study are included in this published article.

## Abbreviations

AFP: Alpha-fetoprotein
CT: Computed tomography
DDLT: Deceased donor liver transplantation
GTV: Gross tumor volume
HCC: Hepatocellular carcinoma
LT: Liver transplantation
LDLT: Living donor liver transplantation
MRI: Magnetic resonance imaging
mRECIST: Modified Radiographic Evaluation Criteria in Solid Tumors
OS: Overall survival
PTV: Planning target volume
RILD: Radiation-induced liver disease
RFA: Radiofrequency ablation
RFS: Recurrence-free survival
SBRT: Stereotactic body radiotherapy
TACE: Transarterial chemoembolization
UCSF criteria: Expanded set of criteria proposed by the University of California San Francisco
