# Supplementary material for: Clinical outcome and pathologic correlation of stereotactic body radiation therapy as a bridge to transplantation for advanced hepatocellular carcinoma: a case series
Source: Radiat Oncol. 2021 Jan 14;16:15. doi: 10.1186/s13014-020-01739-5 (PMC7807861; doi:10.1186/s13014-020-01739-5)
Supplement: Supplementary file 1 — Additional file 1: Table S1: Prognostic factors influencing RFS and OS after LT using the Cox proportional hazards model. [file 13014_2020_1739_MOESM1_ESM.docx]

Additional file 1: Table S1: Prognostic factors influencing RFS and OS after LT using the Cox proportional hazards model

| Variables | **RFS** | | **OS** | |
| --- | --- | --- | --- | --- |
|  | Univariable | | Univariable | |
|  | HR (95% CI) | p | HR (95% CI) | p |
| **Before SBRT** |  |  |  |  |
| Age, years (≥60 vs. <60) | 0.302 (0.038-2.398) | 0.258 | 0.290(0.000-13.675) | 0.259 |
| Sex (male vs. female) | 2.020(0.255-16.022) | 0.506 | 1.742(0.217-13.998) | 0.602 |
| Etiology |  |  |  |  |
| HBV | 0.172(0.015-1.910) | 0.152 | 0.168(0.015-1.875) | 0.147 |
| HCV | 0.129(0.006-2.788) | 0.191 | 0.160(0.008-3.389) | 0.239 |
| Alcohol |  | 0.334 |  | 0.344 |
| Tumor size (≥5cm vs. <5cm) | 0.949(0.271-3.324) | 0.935 | 0.867(0.231-3.248) | 0.832 |
| No of tumors (Multiple vs solitary) | 3.019(0.593-15.354) | 0.183 | 1.887(0.385-9.242) | 0.434 |
| Portal vein thrombosis | 0.794(0.203-3.112) | 0.794 | 0.903(0.222-3.666) | 0.886 |
| Stage, AJCC (III–IV vs. I–II) | 0.736(0.187-2.897) | 0.661 | 0.510(0.104-2.490) | 0.405 |
| BCLC stage (C–D vs. 0–B) | 0.633(0.163-2.460) | 0.509 | 0.740(0.184-2.971) | 0.671 |
| Child-Pugh class (B-C vs. A) | 0.472(0.060-3.743) | 0.478 | 0.599(0.075-4.807) | 0.629 |
| ECOG (1-2 vs. 0) | 1.055(0.271-4.108) | 0.939 | 0.644(0.133-3.123) | 0.585 |
| No. of previous treatments |  | 0.220 |  | 0.646 |
| 1-2 vs. 0 | 3.222(0.388-26.723) | 0.278 | 1.685(0.199-14.275) | 0.632 |
| ≥2 vs. 0 | 0.904(0.810-10.029) | 0.934 | 0.821(0.073-9.219) | 0.873 |
| AFP (≥20 vs. <20 IU/mL) | 0.482(0.134-1.742) | 0.266 | 0.404(0.100-1.633) | 0.240 |
| Milan criteria before SBRT (beyond vs. within) | 1.196(0/252-5.690) | 0.822 | 0.968(0.197-4.744) | 0.968 |
| EQD2 | 0.989(0.957-1.021) | 0.493 | 1.003(0.970-1.037)) | 0.875 |
| **After SBRT** |  |  |  |  |
| SBRT response (SD-PD vs. CR-PR) | 0.659(0.169-2.576) | 0.549 | 0.405(0.082-1.990) | 0.266 |
| Recurrence before LT | 1.513(0.319-7.185) | 0.602 | 1.560(0.322-7.550) | 0.580 |
| Time interval to LT | 1.031(0.990-1.074) | 0.141 | 1.042(0.998-1.087) | 0.059 |
| Pathological necrosis % | 0.922(0.975-1.009) | 0.358 | 0.993(0.976-1.009) | 0.391 |
| Milan criteria before LT (beyond vs. within) | 0.877(0.222-3.471) | 0.852 | 0.722(0.176-2.966) | 0.651 |

Abbreviations: AFP = Alpha-fetoprotein; AJCC = American Joint Committee on Cancer; BCLC = Barcelona Clinic Liver Cancer; CI = confidence interval; CR = complete response; CTP = Child-Turcotte-Pugh class; ECOG = Eastern Cooperative Oncology; EQD2 = Equivalent dose in 2Gy fractions; HBV = hepatitis B virus; HCV = hepatitis C virus; HR = hazard ratio; LT = liver transplantation, No = number; OS = overall survival; PD = progressive disease; PR = partial response; RFS = recurrence free survival, SBRT = stereotactic body radiation therapy; SD = stable disease
